# Prefrontal serotonin depletion delays reversal learning and increases theta synchronization of the infralimbic-prelimbic-orbitofrontal prefrontal cortex circuit

**DOI:** 10.3389/fphar.2024.1501896

**Published:** 2024-12-03

**Authors:** Yoana Estrada-Reyes, José Miguel Cervantes-Alfaro, Miguel Ángel López-Vázquez, María Esther Olvera-Cortés

**Affiliations:** ^1^ Laboratorio de Neuroplasticidad de los Procesos Cognitivos, Centro de Investigación Biomédica de Michoacán, Instituto Mexicano del Seguro Social, Morelia, Michoacán, Mexico; ^2^ Laboratorio de Neurofisiología Clínica y Experimental, Centro de Investigación Biomédica de Michoacán, Instituto Mexicano del Seguro Social, Morelia, Michoacán, Mexico; ^3^ Laboratorio de Neurociencias, Departamento de Posgrado, Facultad de Ciencias Médicas Y biológicas Dr. Ignacio Chávez, Universidad Michoacana de San Nicolás de Hidalgo, Morelia, Michoacán, Mexico

**Keywords:** spatial learning, reversal learning, serotonin, theta activity, prefrontal Cortex

## Abstract

**Introduction:**

Prefrontal serotonin plays a role in the expression of flexible behavior during reversal learning tasks as its depletion delays reversal learning. However, the mechanisms by which serotonin modulates the prefrontal cortex functions during reversal learning remain unclear. Nevertheless, serotonin has been shown to modulate theta activity during spatial learning and memory.

**Methods:**

We evaluated the effects of prefrontal serotonin depletion on theta activity in the prefrontal infralimbic, prelimbic, and orbitofrontal (IL, PL, and OFC) subregions of male rats during a spatial reversal learning task in an aquatic T-maze.

**Results:**

Prefrontal serotonin depletion delayed spatial reversal learning and increased theta activity power in the PL and OFC. Furthermore, animals with serotonin depletion had increased functional coupling between the OFC and the IL and PL cortices compared with the control group.

**Discussion:**

These results indicate that serotonin regulates reversal learning through modulation of prefrontal theta activity by tuning both the power and functional synchronization of the prefrontal subregions.

## 1 Introduction

The serotonergic system is involved in the modulation of reversal learning, a cognitive task in which behavioral flexibility is assessed, through its role as a modulator of prefrontal cortex (PFC) functioning (Alsio et al., 2021; Clarke et al., 2004; Clarke et al., 2007; Man et al., 2010). Reversal learning requires the learning of a first response to an environmental condition and the inhibition of that response with change in the condition. Thus, behavioral flexibility implies learning of a new response to changes in environmental conditions (Izquierdo and Jentsch, 2012; Schiller and Delgado, 2010).

The contribution of each PFC subregion to behavioral flexibility differs depending on the type of task used for its evaluation (Schiller and Delgado, 2010; Dias and Aggleton, 2000; Izquierdo et al., 2017; Ragozzino, 2007). For example, Chudasama and Robbins (2003) showed that damage to the infralimbic (IL) cortex delayed the reversal of a visual discrimination task, whereas Ashwell and Ito (2014) reported that inactivation of this subregion facilitated the reversal of a spatial learning task. Likewise, Dalton et al. (2016) showed that inactivation of the prelimbic (PL) cortex facilitates the reversal of probabilistic learning. However, simultaneous inactivation of the IL and PL cortices does not affect reversal learning, although it does affect changes in multidimensional or trans-modal attentional sets (Ragozzino et al., 1999; Shaw et al., 2013; Young and Shapiro, 2009). The damage or inactivation of the orbitofrontal cortex (OFC) causes a delay in reversal learning tests but does not affect the change in the attentional setting (Ragozzino, 2007; Dalton et al., 2016; Young and Shapiro, 2009; Boulougouris et al., 2007; Chudasama and Robbins, 2004; Stalnaker et al., 2007).

The PFC is densely innervated by dorsal raphe serotonergic neurons (Goncalves et al., 2009), and depletion of cerebral serotonin (5-HT) in rodents and human beings does not show any effect on initial learning in the reversal learning tasks; however, cognitive behavioral performance showing the retainment of the first learned response delayed reversal learning (Alsio et al., 2021; Kanen et al., 2021; Roberts, 2011; Zhukovsky et al., 2017; Colgin, 2013). Additionally, in non-human primates and rats, the specific depletion of 5-HT in the OFC delays reversal learning of serial discriminations, without affecting the learning of discriminations (Alsio et al., 2021; Clarke et al., 2004; Clarke et al., 2007). In contrast, Alsio et al. (2021) observed that depletion of 5-HT in the medial prefrontal cortex (mPFC) increased the number of sessions required to learn visual discrimination without a subsequent reversal effect. This evidence supports the role of prefrontal 5-HT in modulating reversal learning; however, the neurophysiological processes modulated by 5-HT during reversal learning remain to be investigated. 5-HT has been implicated in the modulation of theta activity (4–12 Hz), a type of oscillatory activity that correlates with learning and memory in different cognitive tasks (Colgin, 2013; Gutierrez-Guzman et al., 2011; Gutierrez-Guzman et al., 2012; Gutierrez-Guzman et al., 2017; Lopez-Vazquez et al., 2014; O'Neill et al., 2013; Paz et al., 2008). In this regard, Gutierrez-Guzman et al. (2011) observed that 5-HT depletion in the hippocampus facilitated the learning of a spatial memory task and increase in high-frequency theta activity recorded during the test. Similarly, 5-HT depletion in the medial septal region facilitated the learning of a working memory task and increased high-frequency theta activity (Lopez-Vazquez et al., 2014). Taken together, the previous evidence suggested that 5-HT modulates theta activity in the PFC during reversal learning. Thus, the aim of this study was to evaluate the effect of 5-HT depletion in the prefrontal subregions (IL, PL, and OFC) on the modulation of reversal learning and their correlation with the characteristics of the underlying theta activity.

## 2 Materials and methods

Thirty-one male Sprague–Dawley rats weighing between 350 and 450 g were used in this study. All rats were maintained under standard vivarium conditions: regular 12/12 h light–dark cycle, constant room temperature at 23°C, with *ad libitum* access to water and food. The experiments were performed in accordance with the National Institutes of Health Guide regarding the Care and Use of Laboratory Animals (NIH Publication no. 80-23) and with the “Norma Oficial Mexicana para el uso de animales de laboratorio” (NOM-062-ZOO-1999). Adequate previsions were taken to use minimal number of animals, according to the experimental design, as well as to minimize the animal’s pain or discomfort.

### 2.1 Apparatus

An aquatic T-maze filled with dark stained water (length: 62 cm main arm, two 54 cm side arms; width: 15 cm between walls; height 62 cm), with mobile doors allowing the rat’s displacement from the main arm to either of the side arms or turning back and an escape platform (5 cm × 6 cm surface area and 29 cm high) hidden 3 cm under dark water at the end of the lateral arms, was used for cognitive behavioral testing. The maze was placed in a room, into a Faraday cage with environmental visual signals. A commutator above the maze enabled cable connections between chronically implanted electrodes, in the rat’s head, and the recording equipment in order to obtain recordings of field electric activity from several brain regions, which were stored in a computer, during the different steps of the cognitive behavioral tests.

### 2.2 Surgery

Under general anesthesia (pentobarbital, 60 mg/kg, ip), the rats randomly allotted to the experimental group (n = 16) received an intracerebral injection of 8 µg of 5,7-dihydroxytryptamine creatinine sulfate salt (5,7-DHT) dissolved in 0.5 µL of saline solution with 0.1% of ascorbic acid (as an antioxidant) in the PL cortex (2.7 mm anterior to the bregma, ±0.5 mm lateral, and 2.6 mm dorsoventral), IL cortex (2.7 mm anterior to the bregma, ±0.5 mm lateral, and 3.6 mm dorsoventral), and 1.0 µL in the OFC (4.0 mm anterior to the bregma, ±2.0 mm lateral, and 3.5 mm dorsoventral), according to stereotaxic coordinates of the Atlas of Paxinos and Watson (Paxinos and Watson, 1997). The micro-infusions were carried out through a stereotaxic-oriented 10-µL/26s-gauge Hamilton syringe, at a diffusion rate of 0.1 μL/min. Intraperitoneal desipramine (DMI, 30 mg/kg) was administered to the experimental group rats, 30 min before 5,7-DHT infusion, in order to avoid its neurotoxicity on noradrenergic synaptic terminals. The rats in the control group (n = 15) received an intracerebral injection of saline solution at placements, volumes, and rates similar to those received by the 5,7-DHT experimental group.

During the same surgical performance, bipolar concentric electrodes (insulated 60-µm nichrome wire cemented inside a 27G stainless steel cannula insulated with epoxy resin, with a small recording surface left exposed at the tip of the barrel) were stereotaxically placed in the PL cortex (3.1 mm anterior to the bregma, −0.5 mm lateral, and 3.4 dorsoventral), IL cortex (3.1 mm anterior to the bregma, −0.5 mm lateral, and 5.1 mm dorsoventral), and OFC (3.5 mm anterior to the bregma, −3.0 mm lateral, and 5.0 mm dorsoventral) (Paxinos and Watson, 1997). A stainless-steel screw was placed in the frontal bone and used as the ground electrode. The recording and ground electrodes were assembled in a connector that was firmly fixed to the skull using dental acrylic.

### 2.3 Spatial reversal learning task

**FIGURE 1:**
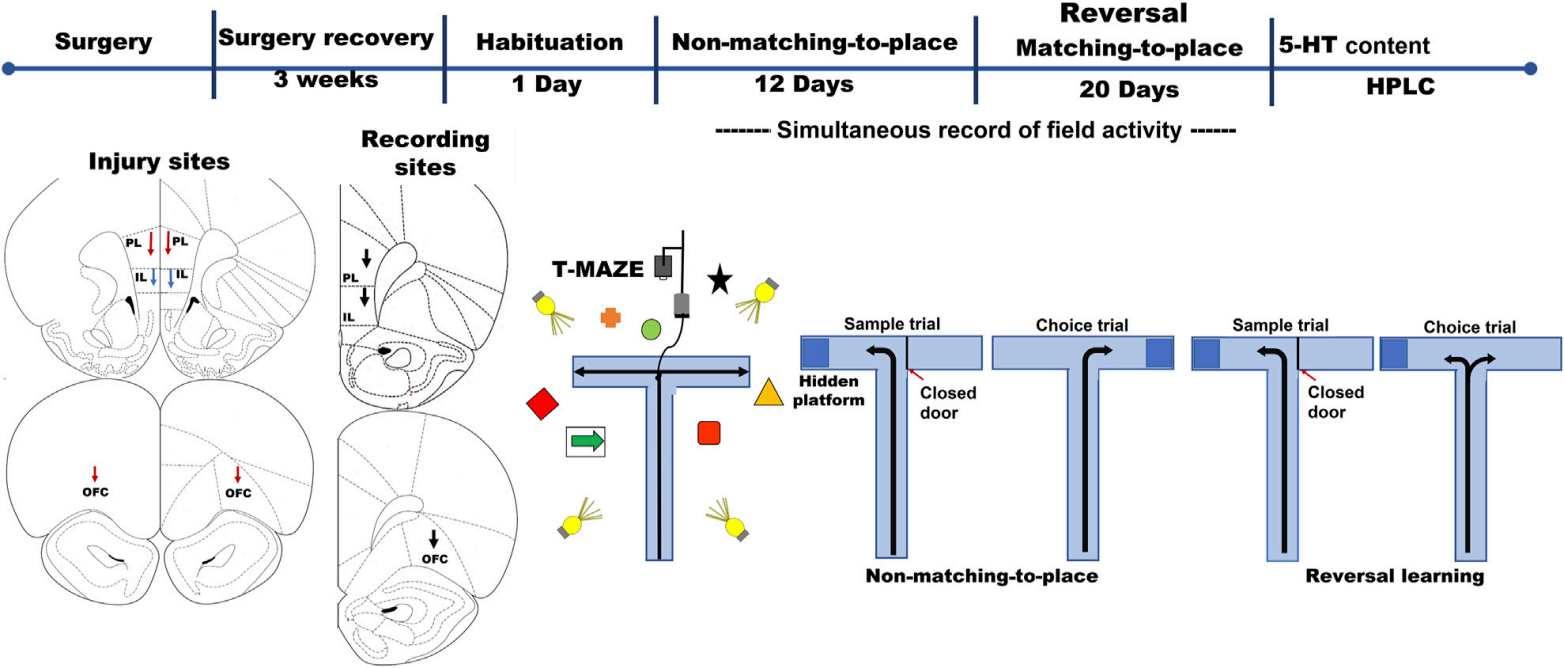
Top: timeline depicting the experimental process. After 3 weeks of recovery from the surgery to infuse 5,7-DHT and to implant the electrodes, the rats were required to learn a reversal learning test: a first rule (non-matching-to-position) was learned in 12 consecutive days with ten trials per day. The second rule (matching-to-position, reversal learning test) was trained for 20 consecutive days, with ten trials per day. A graphic representation of the maze and spatial cue array is shown in the bottom right panel, along with a sample of one trial of each rule. The diagram showing the lesion and recording sites is presented in the bottom left panel.

After 3 weeks of recovery from the surgery, the rats were habituated to the experimental environment through free swimming during 1 min into the maze (Figure 1). The next day, the rats underwent a daily session training period until they achieved a learning criterion, setting on 70% of correct responses, following the non-matching-to-place rule, each daily training session, on 2 consecutive days. Each daily training session consisted of 10 pairs of sample test trials, with a 15-s intra-trial interval and 30-s intervals between pairs of trials (gently placing the rats in a housing cage). Each pair of trials began with the sample trial, in which the rat was placed to the maze facing the wall of the main arm with access to only one lateral arm, where the escape platform was placed at the end (forced election). The rat was permitted to remain on the platform for 15 s; immediately after, the rat was removed and placed in the housing cage for 15 s (delay period), and then each rat was placed again in the central arm facing the wall, with free access to both lateral arms (test trial). In the non-matching-to-place rule (first rule), the escape platform was placed in the arm opposite to the open arm used in the sample trial. The platform position was distributed in a pseudo-random fashion, five trials in the left arm and five in the right arm. Different arrangements of the platform position were used each training day, and through each training day, all rats were tested under the same platform position sequence. Correct and incorrect responses made in each training session during the test trials were counted. In addition, the latency to choose the arm (from the rat being released into the long arm until it enters all the body in the chosen arm) was recorded. The learning criterion was set to 70% of correct responses per session for 2 consecutive days. Once the non-matching-to-place rule was learned, a training period was initiated to learn the reversal task, in which the animals learned the opposite matching-to-place rule, along daily sessions (10 pairs of sample test trials, with an intra-trial interval of 15 s and an interval between pairs of trials of 30 s, during which the animals were placed in an external maintenance cage); however, in each daily task, the platform in the test trial was placed in the same side arm, as shown in the sample trial. Training in the reversal learning task was performed until the animals reached 70% of efficiency in two consecutive daily sessions. During the test trials in which the rats entered the wrong arm, the door allowing access to the side arm was closed, so the rats were confined to the side arm without an escape possibility for 15 s (as punishment) before being taken to the housing cage.

### 2.4 Recording of local field activity

The prefrontal electrical field activity was simultaneously recorded during the behavioral cognitive performance along different steps of training sessions. Local electric field activity signal recordings from the IL, PL, and OFC were obtained at the baseline condition (taken from the wet awake and immobile rats in a housing cage for 30 s before the training trials, once a day), as well as during the performance of each (sampling and testing) trial in the maze (taken since the rat was released into the long arm of the T maze facing the wall until it entered swimming to the chosen arm with all its body), during the delay period (15 s in the housing cage between the sample and test trials), during the 15 s on the escape platform, or during the 15 s of punishment (if this was the case) in each trial in the T-maze.

The signal recordings were stored for offline analysis with a sampling frequency of 1,024 Hz (DataWave Technologies data acquisition system), the band-pass filter was set to 1–100 Hz, and a notch filter at 60 Hz was applied to eliminate line noise. The recorded signals were analyzed by fast Fourier transform using the EEGLAB application in MATLAB. The absolute power was obtained as the mean spectrum of 1-s samples of each behavioral condition (baseline, sample trials, and choice trials), with a resolution of 0.5 Hz, and the absolute power values were transformed into natural logarithms (Ln). The coherence between prefrontal cortex subregions (IL-PL, IL-OFC, and PL-OFC) was calculated as the squared magnitude of coherence Cxy (f) by using a modified periodogram method. Three representative blocks of learning training days were selected for each task. In the task of not matching to the place, the blocks chosen were as follows: B1, corresponding to sessions 1 and 2 (initial phase); B4, which included sessions 7 and 8 (intermediate phase when the correct responses reached 50%); and B6, which included sessions 11 and 12 (end of the task) when the rats achieved 70% of correct responses. For the reversal task, the blocks were as follows: B1, corresponding to sessions 1 and 2 (initial phase, when the rats responded under the previous rule); B6, which corresponds to days 11 and 12, when the rats had an efficiency of 50% correct responses; B8, corresponding to sessions 15 and 16, in which the control group achieved the learning criterion (70%); and finally, B10, which corresponds to sessions 19 and 20, when the responses of the control group were stable. In these blocks, the mean power spectrum and mean coherence were obtained from all choice trials (20 choice trials).

### 2.5 Neurochemical assessment

The day after the last behavioral cognitive trial, showing achievement of learning criterion (70% correct responses) of the matching-to-place rule, the rats were euthanized, the brain was promptly removed, and the orbitofrontal and medial prefrontal cortex tissues were carefully dissected from brain slices and placed in a glass iced surface. The brain tissue was simultaneously obtained from four-rat sets belonging to the control and 5-HT groups. The tissue was homogenized with 0.1 N hydrochloric acid (HCl), washed with deionized and filtered water, and perchloric acid was added, followed by centrifugation for 5 min at 5,000 rpm. Serotonin (5-HT), 5-hydroxy-indole-acetic acid (5HIAA), dopamine (DA), and 3,4-dihydroxyphenylacetic acid (DOPAC) levels (pg/mg fresh tissue) were measured using a LiChroCART Purospher Star column (150 × 4.6, RP-18 end-capped, 5 mm, MERK Kga A, Darmstadt, Germany). The mobile phase was composed of citric acid (50 mM), H_3_PO_4_ (50 mM), EDTA (20 mg), octanesulfonic acid (120 mg/L), and methanol (7%) at pH 3.1, with a flow rate of 1.3 mL/min. Catecholamine amounts (pg/gm) in control sets were taken as 100% in order to calculate the percentage of serotonin and 5HIAA reduction in the 5-HT sets and the DA and DOPAC content.

### 2.6 Data analysis

The efficiency of the animals in solving behavioral tasks was analyzed based on the average number of correct responses in the test trial per block, where each block corresponded to the behavioral responses of 2 consecutive days of training. The number of correct responses and the latencies to choice arm in the test trials were compared using repeated-measures analysis of variance, and the t-test with *post hoc* Bonferroni adjustment was applied. For power and coherence, repeated-measure analysis of variance for three factors (group, condition, and block) was performed, and Tukey’s *post hoc* test was applied. Comparisons were made between the different stages (baseline, correct, and incorrect responses) per block (mean of two consecutive training sessions) and between the different blocks of the same stage. Statistical significance was set at *p* < 0.05.

## 3 Results

**FIGURE 2:**
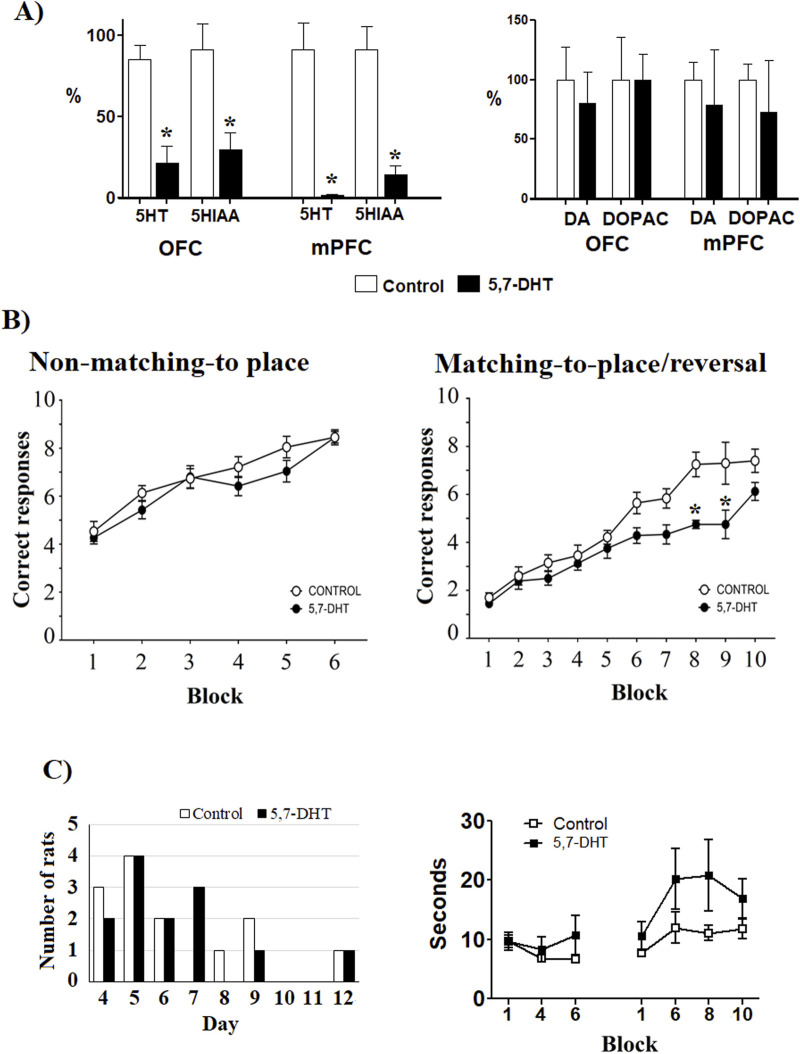
**(A)** Left: prefrontal serotonin levels. The levels of 5HT and 5-HIAA (percentage from control) in the medial prefrontal cortex (PL and IL cortices) and the orbitofrontal cortex (OFC) were measured by HPLC. Mean ± S.E.M. *group control vs 5,7-DHT. Right: dopamine and DOPAC content in OFC and mPFC; no significant differences were observed. Mean ± S.E.M. **(B)** Left: performance in the aquatic T-maze. In the non-matching-to-place test, no differences were observed between groups in correct trials. Right: correct responses of the reversal learning test. The 5,7-DHT group failed to meet the reversal learning criterion and had a minor number of correct responses than the control group in blocks 8 and 9 (days 15–18 of training). Mean ± S.E.M. *group control vs 5,7-DHT, *p* < 0.05. **(C)** Left: number of rats that performed 2 consecutive days with seven or more correct responses through the training days in the first rule (non-matching-to-position). No differences in distribution were observed between groups. Right: latencies to choose the arm in the test trials. No significant differences by group and block were observed. Values are expressed as mean ± S.E.M.

Data of cognitive behavioral performance and power and coherence of brain electrical activity obtained from 14 control rats and from 13 rats with 75% depletion of 5-HT in both the mPFC (5-HT: t = 4.157; df = 11, *p* = 0.002; 5HIAA: t = 3.685, df = 13, *p* = 0.003) and OFC (5-HT: t = 2.542, df = 9, *p* = 0.021; 5HIAA: t = 1.901, df = 14 *p* = 0.078), as compared to prefrontal 5-HT levels in control animals (100%), were analyzed (Figure 2A, left). No differences were observed in DA and DOPAC levels between the groups (Figure 2A, right).

### 3.1 Spatial reversal learning

The number of correct and incorrect responses made by the animals during the execution of the behavioral tasks on each day of testing was recorded. The correct responses in the test trials per training session were averaged in blocks of 2 days, and intragroup and inter-group comparisons were made for learning and reversal tasks.

In learning of the non-matching-to-place rule, no significant differences were observed between the groups with respect to the number of correct responses made throughout the training blocks [F (Alsio et al., 2021; Kanen et al., 2021) = 2.507, *p* = 0.129]. These results indicate that prefrontal 5-HT depletion did not affect the ability to learn the first rule (Figure 2B, left). However, 5-HT depletion impaired the progressive learning of the reversal learning task, as shown by intergroup comparisons [F (Alsio et al., 2021; Dias and Aggleton, 2000) = 8.204, *p* = 0.024]. Thus, while the control group reached 70% of correct responses in block 8 (B8, days 15 and 16) and maintained their performance in the following two blocks (B9, days 17 and 18; B10, days 19 and 20), the 5,7-DHT group failed to meet the learning criteria (70% correct responses) through the entire training period, leading to significant differences, between groups, in the number of correct responses attained during the last training blocks (B8, control n = 6, 5,7-DHT n = 6, *p* < 0.001; B9, control n = 5, 5.7-DHT n = 4, *p* = 0.003; B10, control n = 5, 5,7-DHT n = 4, *p* = 0.05) (Figure 2B, right). To determine whether the impaired reversal learning was due to overtraining in the 5,7-DHT group, the number of days required to achieve the learning criteria in the first test was compared. The distribution of rats reaching 70% efficiency for 2 consecutive days during training was similar for both groups (Figure 2C, left). The latencies to enter in the chosen arm did not show significant differences [F (6.90) = 0.8973, *p* = 0.506], although a trend to longer latencies for the 5,7-DHT group was observed (t = 2.074, *p* = 0.60) (Figure 2C, right). From these results, a strong deficiency in spatial reversal learning after 5-HT depletion in the prefrontal circuit was evident.

### 3.2 Electrical field activity in the prefrontal subregions during spatial reversal learning

The absolute power of the theta band (4–12 Hz) in the prefrontal subregions (IL, PL, and OFC) and the coherence between the subregions (IL-PL, IL- OFC, and PL-OFC) during the baseline condition (awake and immobile at the start of the daily training) and during test trials (when the rats made the choice of the arm) for non-matching- (BI, B4, and B6) and matching-to-place (B1, B6, B8, and B10) rules were analyzed. In the analysis of the data, no significant differences were found between correct and incorrect responses in the test trials.

#### 3.2.1 Theta power

##### 3.2.1.1 Intragroup analysis of absolute power in prefrontal subregions

**FIGURE 3:**
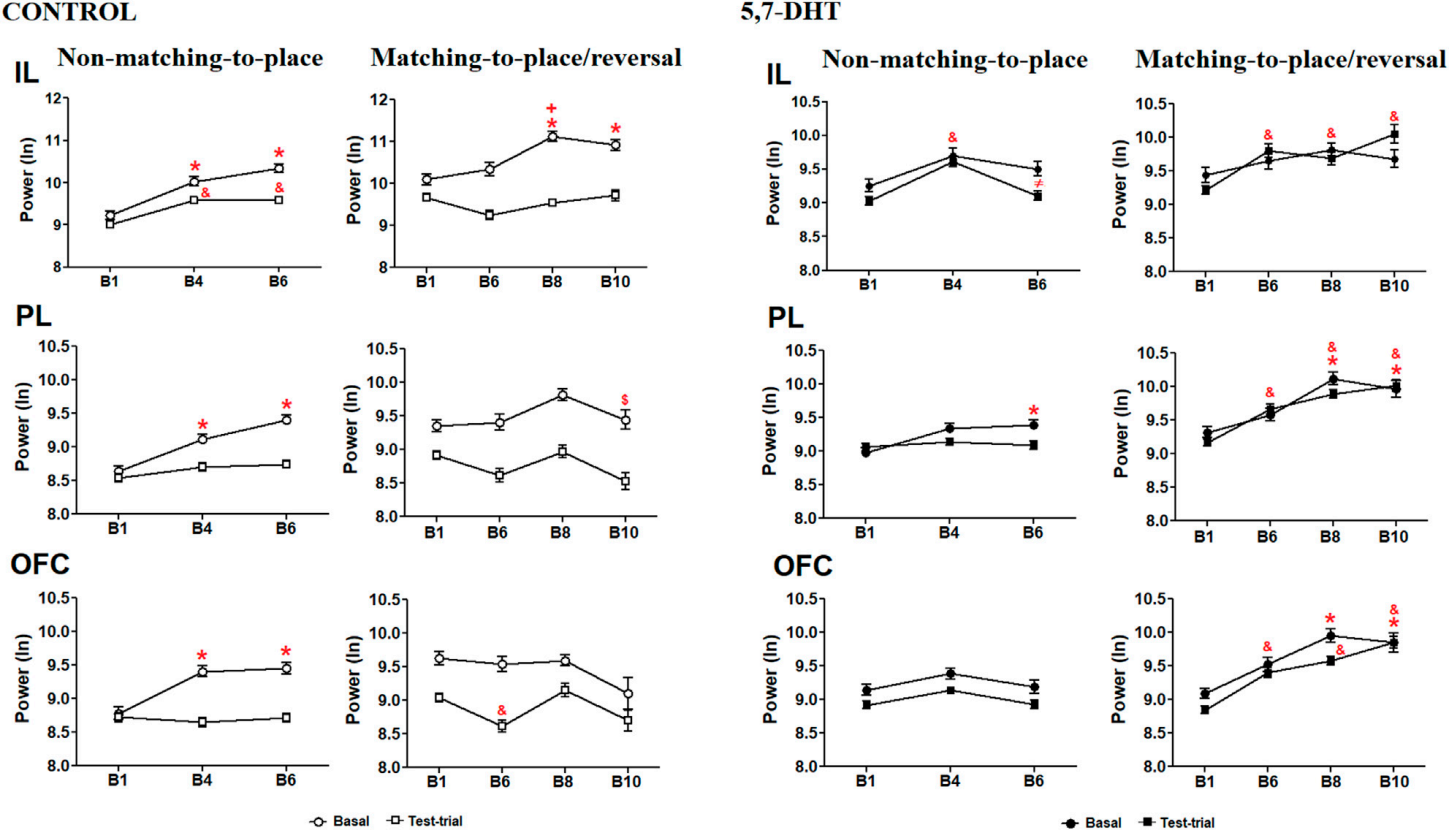
Left: normalized absolute theta power of the prefrontal subregions recorded during the baseline and test trials in the behavioral task of the control group. IL cortex (top), PL cortex (middle), and OFC (bottom) theta power natural logarithm (nL). Mean ± S.E.M. Non-matching-to-position: baseline (circles): *B1 vs other blocks; test trials (squares): &B1 vs other blocks, +B6 vs B8; $ B8 vs B10. *p* < 0.05. Right) Normalized absolute theta power of the prefrontal subregions recorded during the behavioral task of the 5,7-DHT group. IL cortex (top), PL cortex (middle), and OFC (bottom) theta power (natural logarithm). Mean ± S.E.M. Baseline (circles): *B1 vs other blocks. Test trials (squares): &B1 vs other blocks, ≠B4 vs B6. *p* < 0.05.

In the control group, intragroup analysis of the absolute power in the IL cortex indicated a significant difference for the factors condition (baseline and test trials) [F (1.66) = 22.193, *p* < 0.001] and condition × block interaction [F (6,396) = 22.577, *p* < 0.001]. During learning of the non-matching-to-place rule, the baseline power in the IL cortex of the control group increased significantly from B1 to blocks B4 (*p* < 0.001) and B6 (*p* < 0.0001) and similarly during the test trials (B1 vs B4, *p* < 0.0001; B1 vs B6, *p* < 0.0001). In the reversal task, the power at baseline increased significantly in B8 (*p* = 0.0001) and B10 (*p* = 0.0166) compared to that in B1, and also in B8 compared with B6 (*p* = 0.0122), whereas no significant differences were observed in the test trials (Figure 3, left-top). In the PL cortex, significant differences were observed in the condition [F (1.66) = 16.699, *p* < 0.001] and condition × block interaction [F (6,396) = 14.889, *p* < 0.001]. In the acquisition of the non-matching-to-place rule, the power in the PL cortex of the control group in the baseline condition increased significantly in blocks B4 (*p* < 0.0001) and B6 (*p* < 0.0001) compared with the first block, whereas no significant changes were observed in the test trials. In the reversal task, the power at baseline decreased significantly from B8 to B10 (*p* = 0.0162), and no significant changes were observed during the test trials (Figure 3, left-middle). Finally, in the OFC, the analyses indicated a significant difference for condition [F (1.66) = 27.927, *p* < 0.001] and for the condition × block interaction [F (6,396) = 15.292, *p* < 0.001]. During the acquisition of the non-matching-to-place rule, the power in the OFC in the baseline condition significantly increased between the blocks B4 p (<0.0001) and B6 (*p* < 0.0001) and the first block, while no significant changes were observed in the test trials. In the reversal task, the power at baseline did not show significant changes, and during the test trials, it decreased significantly from B1 to B4 (*p* = 0.0110) (Figure 3, left-bottom).

Absolute power intragroup analysis of the 5,7-DHT group showed a significant difference by condition [F (1,151) = 35.766, *p* < 0.001] and condition × block interaction [F (6,906) = 6.459, *p* < 0.001] in the IL cortex. During the acquisition of the non-matching-to-place rule, the power at baseline did not show significant differences; however, during test trials, changes were observed depending on the progress of the test. The power increased in B4 compared to B1 (*p* < 0.0001) and decreased in B6 compared to B4 (*p* = 0.0003). In the reversal task, the power at baseline did not show significant changes, whereas the test trial power increased in blocks 6–10 (B1 vs B6, *p* < 0.0001; B1 vs B8, *p* = 0.0275; and B1 vs B10, *p* < 0.0001) as compared to B1 (Figure 3, right-top). In the PL cortex, the results indicated a significant difference by condition [F (1,151) = 43.713, *p* < 0.001] and condition × block interaction [F (6,906) = 3.058, *p* < 0.001]. During the acquisition of the non-matching-to-place rule, the power at baseline exhibited a significant increase in B6 compared to B1 (*p* = 0.0229), whereas no changes were observed in the test trials. In the reversal learning, the power at baseline increased significantly in B8 (*p* < 0.0001) and B10 (*p* = 0.0162) compared with B1, whereas in the test trials, an increase in power was observed from B6 to B10 relative to B1 (*p* < 0.0001) (Figure 3, right-middle). Finally, in the OFC, the comparisons showed significant differences by condition [F (1,151) = 43.713, *p* < 0.001] and condition × block interaction [F (6,906) = 3.058, *p* < 0.001]. For learning of the non-matching-to-place rule, the power did not change significantly in either baseline or test trial conditions, whereas in the reversal task, the baseline power increased significantly in B8 (*p* < 0.001) and B10 (*p* = 0.0031) compared to B1, and the test trial theta power increased from B6 to B10 compared to B1 (*p* < 0.0001) (Figure 3, right-bottom).

##### 3.2.1.2 Inter group analysis of absolute power in prefrontal subregions

The intergroup comparisons were made with ANOVA for repeated measures with the factors group, condition, frequency, and block. The paired comparisons were centered in the test trials and are shown in the following results.

**FIGURE 4:**
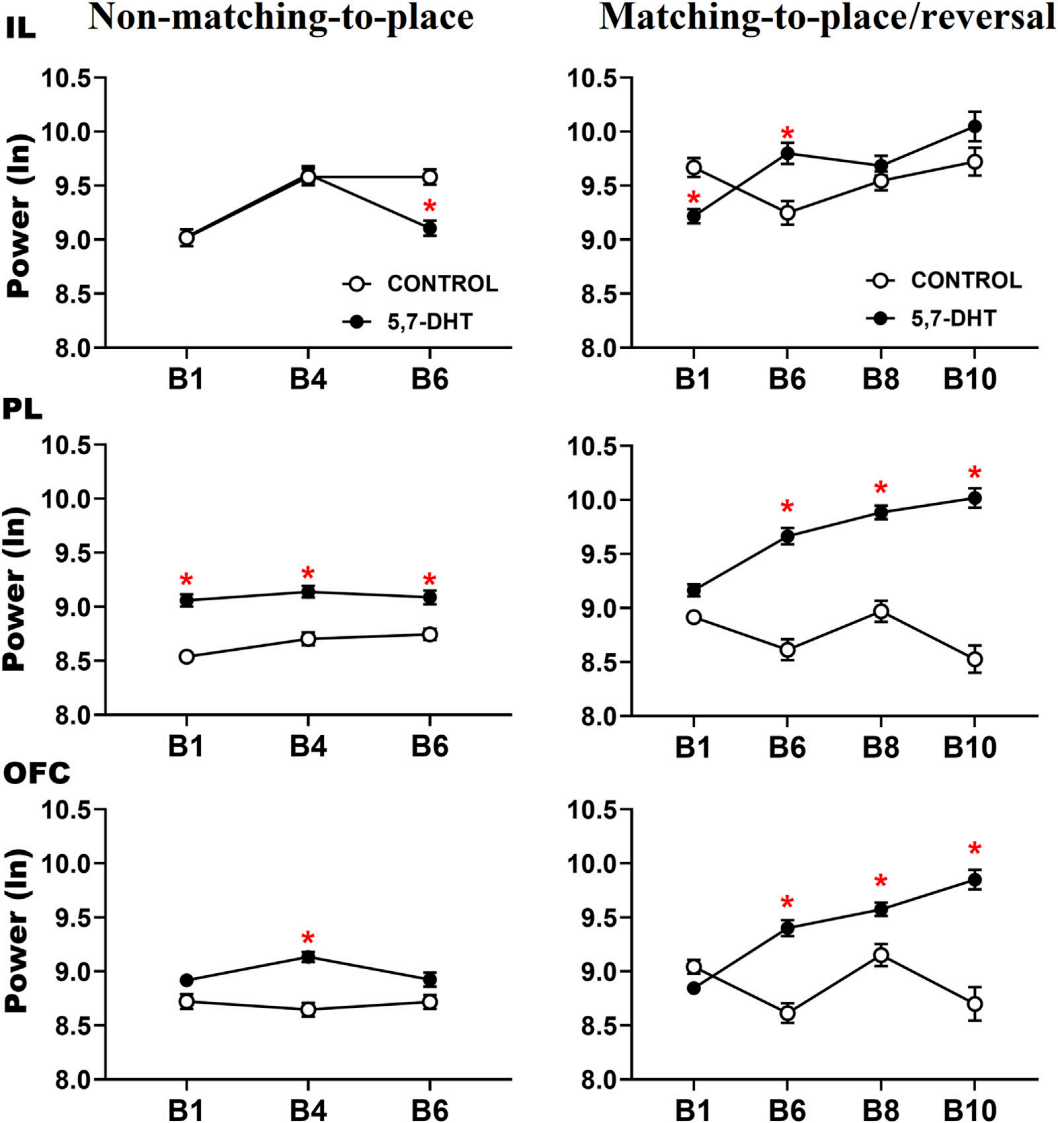
Intergroup comparison of the normalized (natural logarithm, nl) absolute theta power in the prefrontal subregions recorded during the behavioral tasks. IL cortex (top), PL cortex (middle), and OFC (bottom) power. Mean ± S.E.M. *control vs 5,7-DHT. *p* < 0.05.

The intergroup analysis of the absolute power of the IL cortex showed a significant difference between groups [F (1,217) = 566.838 *p* < 0.001] and a significant group × condition interaction [F (1, 217) = 204.685, *p* < 0.001] and group × condition × block interaction [F (6, 1,302) = 11.058, *p* < 0.001]. During learning of the non-matching-to-place rule, the IL power of the 5,7-DHT group was significantly lower than that of the control group at B6 (control group, n = 6 vs 5,7-DHT group, n = 5, *p* = 0.0024). For the inversion task, the power of the 5,7-DHT group was lower in the B1 trial and higher in the B6 trial (control, n = 9 vs 5,7-DHT, n = 9; B1, *p* = 0.0160; B6, *p* = 0.0068) compared to control power (Figure 4, top).

In the PL cortex, power analysis showed a significant group effect [F (1, 217) = 19.509 *p* < 0.001] and a significant group × condition interaction [F (1,217) = 220.186, *p* < 0.001] and group × condition × block interaction [F (6, 1,302) = 5.048, *p* < 0.001]. The decrease in serotonin levels in the 5,7-DHT group increased PL theta power. Regarding learning of the non-matching-to-place rule, no significant differences were observed during the baseline condition. However, in the test trials, the PL power of the 5,7-DHT group was significantly higher than that in the control group in B1 (control, n = 12; 5,7-DHT, n = 10, *p* < 0.0001), B4 (control, n = 13; 5,7-DHT, n = 13, *p* = 0.0001), and B6 (control, n = 10; 5,7-DHT, n = 10, *p* < 0.0093). For the reversal task, no intergroup differences were observed at baseline; however, during the test trials, the power of the 5,7-DHT group was higher than that of the control group from B6 to B10 (B6: control, n = 6; 5,7-DHT, n = 6, *p* < 0.0001; B8: control, n = 5; 5,7-DHT, n = 6, *p* < 0.001; B10: control, n = 4; 5,7-DHT, n = 4, *p* < 0.001) (Figure 4, middle).

In the OFC, the power comparisons showed a significant group effect [F (1,217) = 105.832, *p* < 0.001] and a significant group × condition interaction [F (1, 217) = 110.714, *p* < 0.001] and group × condition × block interaction [F (61,302) = 5.048, *p* < 0.001]. In learning the non-matching-to-place rule, no significant differences were observed in the baseline; however, the power of the 5,7-DHT group was significantly higher than that of the control group in the B4 test trials (control, n = 13; 5,7-DHT, n = 13, *p* = 0.0001). In the reversal task, the power of the 5,7-DHT group was greater than that observed in the control group at baseline in B4 (control, n = 4; 5,7-DHT, n = 4, *p* = 0.0395) and in test trials at B6 (control, n = 6; 5.7-DHT, n = 7, *p* < 0.0110), B8 (control, n = 5; 5,7-DHT, n = 6, *p* < 0.0467), and B10 (control, n = 4; 5,7-DHT, n = 4, *p* < 0.0001) (Figure 4, bottom).

#### 3.2.2 Prefrontal theta coherence

##### 3.2.2.1 Intragroup analysis of coherence in prefrontal subregions

The functional correlation between the prefrontal subregions and the cognitive behavioral performance was determined through the interregional coherence analysis under different experimental conditions and cognitive tasks.

**FIGURE 5:**
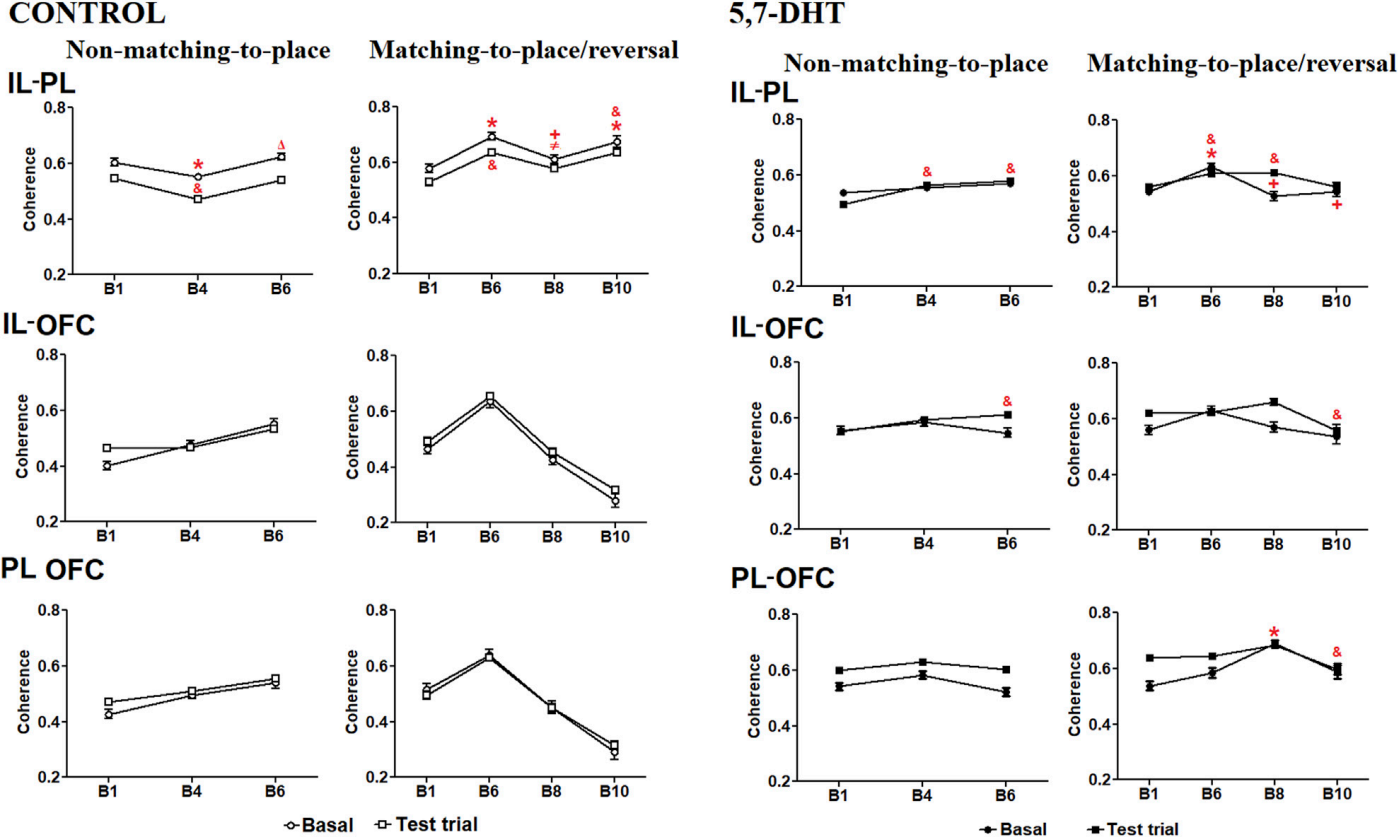
Left: theta coherence of prefrontal subregions during behavioral tasks in the control group. IL-PL coherence (top), IL-OFC coherence (middle), and PL-OFC theta coherence (bottom). Mean ± S.E.M. Baseline: *B1 vs other blocks, ΔB4 vs B6, and +B6 vs B8. Test trials: $B1 vs other blocks. ≠B6 vs B8. (*p* < 0.05. *p* < 0.05. Right: theta coherence between prefrontal subregions during behavioral tasks in the 5,7-DHT group. IL-PL theta coherence (top) increases during the tests. IL-OFC theta coherence (middle) and PL-OFC theta coherence (bottom). Mean ± S.E.M. Baseline: *B1 vs other blocks. Test trials: & B1 vs other blocks, +B6 vs B8 and B10. *p* < 0.05.

The intragroup IL-PL coherence analysis in the control group showed a significant effect of condition [F (1.66) = 31.915, *p* < 0.001] and condition × block interaction [F (6,396) = 8.631, *p* < 0.001] (Figure 5, left-top). During learning of the non-matching-to-place rule, the coherence between the IL-PL in the baseline condition decreased from B1 to B4 (*p* = 0.0291) and increased from B4 to B6 (*p* = 0.0009). The coherence of the test trials decreased from B1 to B4 (*p* < 0.0001), and a posterior increase from B6 to B1 values occurred. In the reversal task, the coherence under the baseline condition changed with the progress of the task; it increased significantly in B6 (*p* = 0.0001) and B10 (*p* = 0.008) compared to B1 and decreased from B6 to B8 (*p* = 0.0139). Similarly, during the test trials, the coherence increased significantly in B6 and B10 compared to B1 (*p* < 0.0001) and decreased from B6 to B8 (*p* = 0.0333).

In the IL-OFC coherence comparisons, the control group analysis did not show a significant effect of condition [F (1.66) = 1.470, *p* = 0.230] or the condition × block interaction [F (6,396) = 1.058, *p* = 0.388]. Significant differences were observed only for the block [F (6,396) = 6.075, *p* < 0.001] (Figure 5, left-middle). The PL-OFC coherence analysis did not show a significant effect of condition [F (1.66) = 1.470, *p* = 0.230] or the condition × block interaction [F (6,396) = 1.058, *p* = 0.388]; there was a significant effect of block [F (6,396) = 6.075, *p* < 0.001] (Figure 5, left-bottom).

Intragroup coherence analysis of the 5,7-DHT group in the IL-PL cortices indicated a significant effect of the condition × block interaction [F (6,906) = 3.613, *p* = 0.002] (Figure 5, right-top). During learning of the non-matching-to-place rule, the coherence at baseline did not change; however, the test trials showed increased coherence in blocks 4 and 6 compared with B1 (*p* < 0.0001). In the reversal task, baseline coherence increased significantly in B6 compared to B1 (*p* = 0.0005) and subsequently decreased in B8 (*p* = 0.0006) and B10 (*p* = 0.022) compared to B6. During the test trials, coherence was significantly higher in B6 (*p* = 0.147) and B8 (*p* = 0.0097) blocks than in the B1.

The intragroup analyses of IL-OFC coherence showed a significant effect of condition [F (1,151) = 6.313, *p* = 0.013] as well as the condition × block interaction [F (6,906) = 4.698, *p* < 0.001] (Figure 5, right-middle). In learning of the non-matching-to-place rule, baseline coherence did not change; however, coherence in the test trials was higher in B6 than in B1 (*p* < 0.0077). In the reversal task, baseline coherence did not change, whereas in the test trials, it decreased significantly in B10 compared to B8 (*p* = 0.0003).

In the intragroup analysis of PL-OFC coherence, a significant effect was observed in condition [F (1,151) = 6.313, *p* = 0.013] as well as in the condition × block interaction [F (6,906) = 4.698, *p* < 0.001] (Figure 5, right-bottom). In learning of the non-matching-to-place rule, coherence did not show significant changes. In contrast, in the reversal task, baseline coherence significantly increased in B8 compared to B1 (*p* < 0.0001) and decreased during the test trials, with significant reduction in B10 compared to B8 (*p* < 0.0001).

##### 3.2.2.2 Inter-group analysis of coherence in prefrontal subregions

**FIGURE 6:**
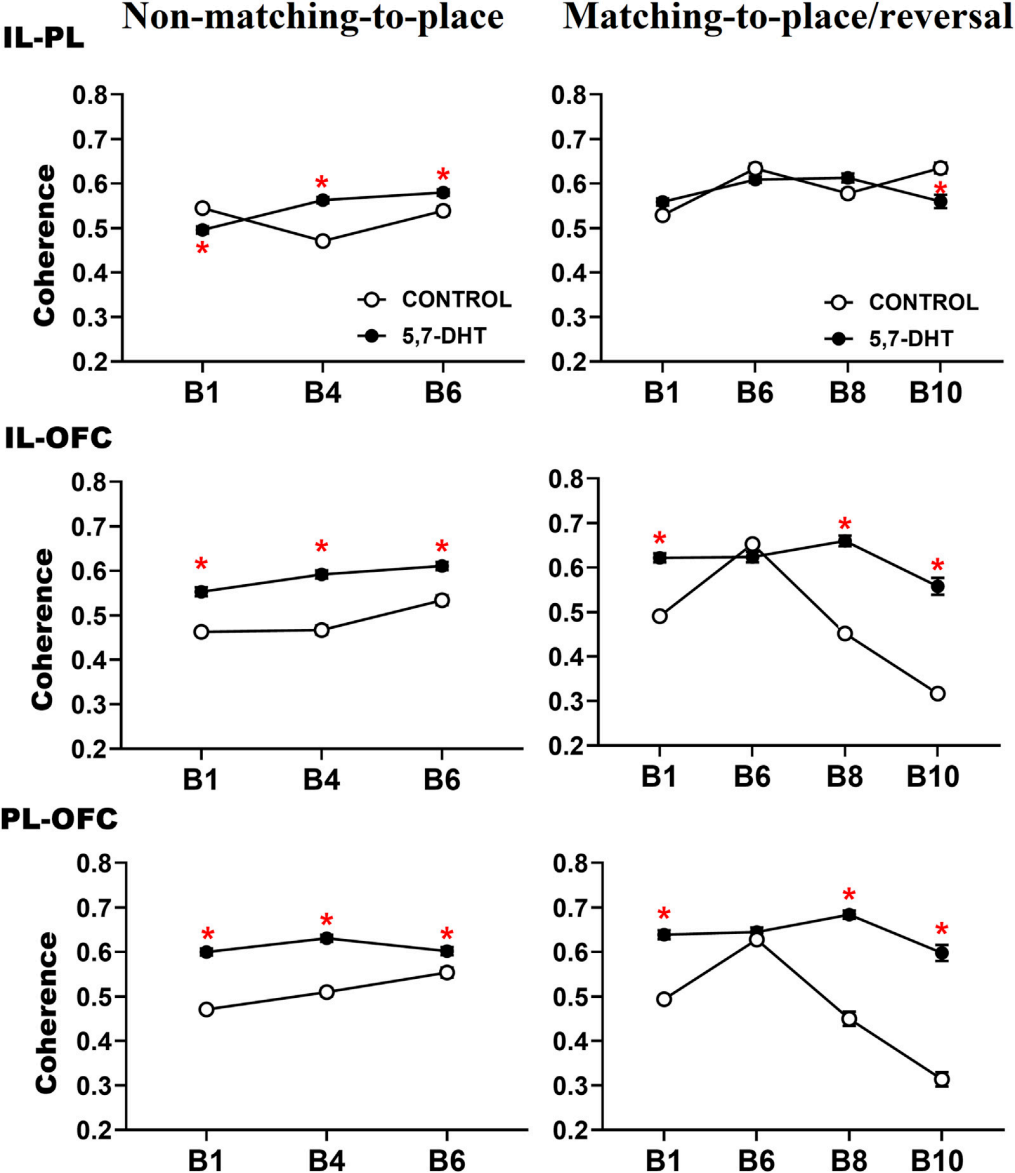
Intergroup comparison of prefrontal subregion theta coherence recorded during the behavioral tasks. The IL-PL theta coherence (top) of the two groups changes depending on their behavioral state. The IL-OFC theta coherence (middle) of the 5,7-DHT group was greater than that of the control group throughout the tasks. The PL-OFC theta coherence (bottom) of the 5,7-DHT group was greater than that of the control group throughout the tasks. Mean ± S.E.M. *group control vs 5,7-DHT.

Similar to power, the paired comparisons of intergroup analysis were centered in test trials. The intergroup comparison of IL-PL coherence did not show a significant per group difference [F (1, 217) = 0.018, *p* = 0.894], whereas the group × condition interaction [F (1, 217) = 5.278, *p* = 0.023] and group × condition × block interaction [F (6, 1,302) = 4.176, *p* < 0.001] showed significant differences. In learning of the non-matching-to-place rule test trials in B1, coherence was lower in the 5,7-DHT group (control, n = 12; 5,7-DHT, n = 10, *p* < 0.001) but higher than that observed in the control group in B4 (control, n = 13; 5,7-DHT, n = 13; *p* < 0.0001) and B6 (control, n = 10; 5,7-DHT, n = 10; *p* = 0.0365). In the reversal task, lower coherence values were observed in the 5,7-DHT group in B10 of the test trials (control, n = 4, 5,7-DHT, n = 4; *p* = 0.0060) (Figure 6, top).

In the intergroup comparisons of IL-OFC coherence, significant effects of group [F (1,217) = 46.789, *p* < 0.001], group × condition interaction [F (1, 217) = 9.776, *p* = 0.002], and group × condition × block interaction [F (6, 1,302) = 4.971, *p* < 0.001] were observed. Paired comparisons showed higher values of coherence between the IL-OFC in the experimental group throughout the behavioral tests. In the non-matching-to-place task, the experimental group had greater coherence values in the three blocks of the test trials: B1 (control, n = 12; 5,7-DHT, n = 10; *p* < 0.0001), B4 (control, n = 13; 5,7-DHT, n = 13; *p* < 0.0001), and B6 (control, n = 10; 5,7-DHT, n = 10; *p* < 0.0001). In the reversal task, the coherence of the 5,7-DHT group was higher in blocks 1, 8, and 10 during the test trials (B1: control, n = 8; 5,7-DHT, n = 9; *p* < 0.0001; B8: control, n = 5; 5,7-DHT, n = 6; *p* < 0.0001; B10: control, n = 4; 5,7-DHT, n = 4; *p* < 0.0001) (Figure 6, middle).

The intergroup PL-OFC coherence comparisons showed a significant effect of group [F (1, 217) = 52.502, *p* < 0.001], group × condition interaction [F (1, 217) = 3.743, *p* = 0.054], and group × condition × block interaction [F (61,302) = 3.283, *p* = 0.003]. The PL-OFC coherence of the 5,7-DHT group was higher than that of the control group throughout the tests, as a general effect of the 5-HT depletion. In the non-matching-to-place rule, a higher coherence was observed in the experimental group in the test trials (B1: control, n = 12; 5,7-DHT, n = 12; *p* < 0.001; B4: control, n = 13; 5,7-DHT, n = 3; *p* < 0.001; B6: control, n = 10; 5,7-DHT, n = 10; *p* < 0.001). In the reversal task, the experimental group showed higher coherence than the control group in blocks 1 (control, n = 8; 5,7-DHT, n = 9; *p* < 0.0001), 8 (control, n = 5; 5,7-DHT, n = 6; *p* < 0.001), and 10 (control, n = 4; 5,7-DHT, n = 4; *p* < 0.0001) of the test trials (Figure 6, bottom).

### 3.3 Theta activity-behavior correlations

**FIGURE 7:**
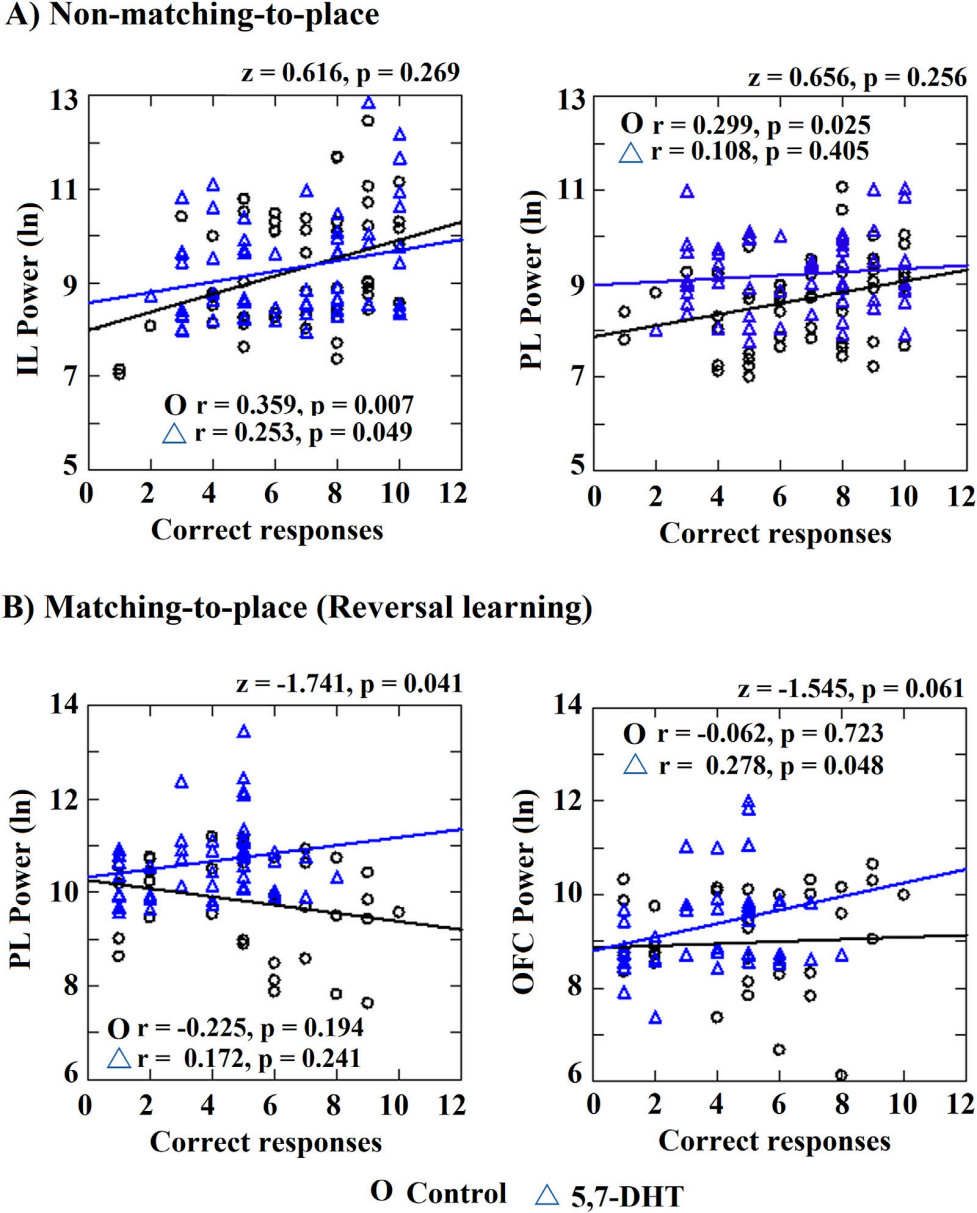
Correlation index between power and number of correct responses. Significant correlations and significantly different between-group correlations are shown. **(A)** Non-matching-to-place IL power and PL power correlations with the number of correct responses. **(B)** Matching-to-place PL and OFC correlations with the number of correct responses. Pearson correlation and differences between correlation index (z values). Circles: control group and triangles: 5,7-DHT group. *p* < 0.05.

The correlation index between the number of correct responses and the theta power recorded from the prefrontal subregions through the study blocks for each of the tasks (matching-to-place and non-matching-to-place tests) was obtained. In the control group, for the non-matching-to-place task, a significant positive correlation between the number of correct responses and the power of the IL (r = 0.359, *p* = 0.007) and PL (r = 0.299, *p* = 0.025) cortices was observed, but no significant correlation was observed in the OFC (r = 0.020 *p* = 0.884) (Figure 7A). Thus, when the animals performed more correct responses, the theta power in the prefrontal subregions was higher and was associated with the success amounted in learning the cognitive behavioral task.

In the 5,7-DHT group, a significant positive relation between the correct responses and the IL theta power was observed (r = 0.253, *p* = 0.049); however, in contrast to the control group, the PL theta power and correct responses correlation in this group was not significant (r = 0.108, *p* = 0.405). Finally, the OFC theta power correlation was not significant (r = 0.187, *p* = 0.150). When comparing the correlation indices between groups, no significant differences were observed for the IL (z = 0.616, *p* = 0.269), PL (z = 0.656, *p* = 0.256) and OFC (z = −0.891, *p* = 0.187).

Regarding the matching-to-place task (reversal task), the control group did not show significant correlations between the correct responses and the theta power of the cortices (IL r = −008, *p* = 0.964; PL r = −0.225, *p* = 0.194; OFC r = −0.062, *p* = 0.723). For its part, the 5,7-DHT group showed a significant positive correlation between correct responses and OFC theta power (r = 0.287, *p* = 0.048), but not to IL (r = 0.137, *p* = 0.353) or PL (r = 0.172, *p* = 0.241). However, when comparing the correlation indices between groups, significant differences were observed in the correlation values of the PL cortex (z = −1.741, *p* = 0.041) because in the control group, the power tends to decrease as the new task is acquired, while in the 5,7-DHT group, a trend for increased power is observed. No differences were observed for IL (z = 0.631, *p* = 0.264) and a trend was observed to OFC (z = −1.545, *p* = 0.061) (Figure 7B).

The correlation between the number of correct responses and the coherence obtained between the pairs of prefrontal subregions (IL-PL, IL-OFC, and PL-OFC) was analyzed. The control group correlation analysis was not significant in the non-matching-to-place task (IL-PL r = −0.155, *p* = 0.259; IL-OFC r = 0.156, *p* = 0.256; PL-OFC r = 0.256, *p* = 0.098). However, the 5,7-DHT group had a non-significant negative correlation between the number of correct responses and the PL-OFC (r = −0.230, *p* = 0.077), IL-PL (r = −0.207, *p* = 0.109), and IL-OFC (r = −0.019, *p* = 0.882) coherence.

**FIGURE 8:**
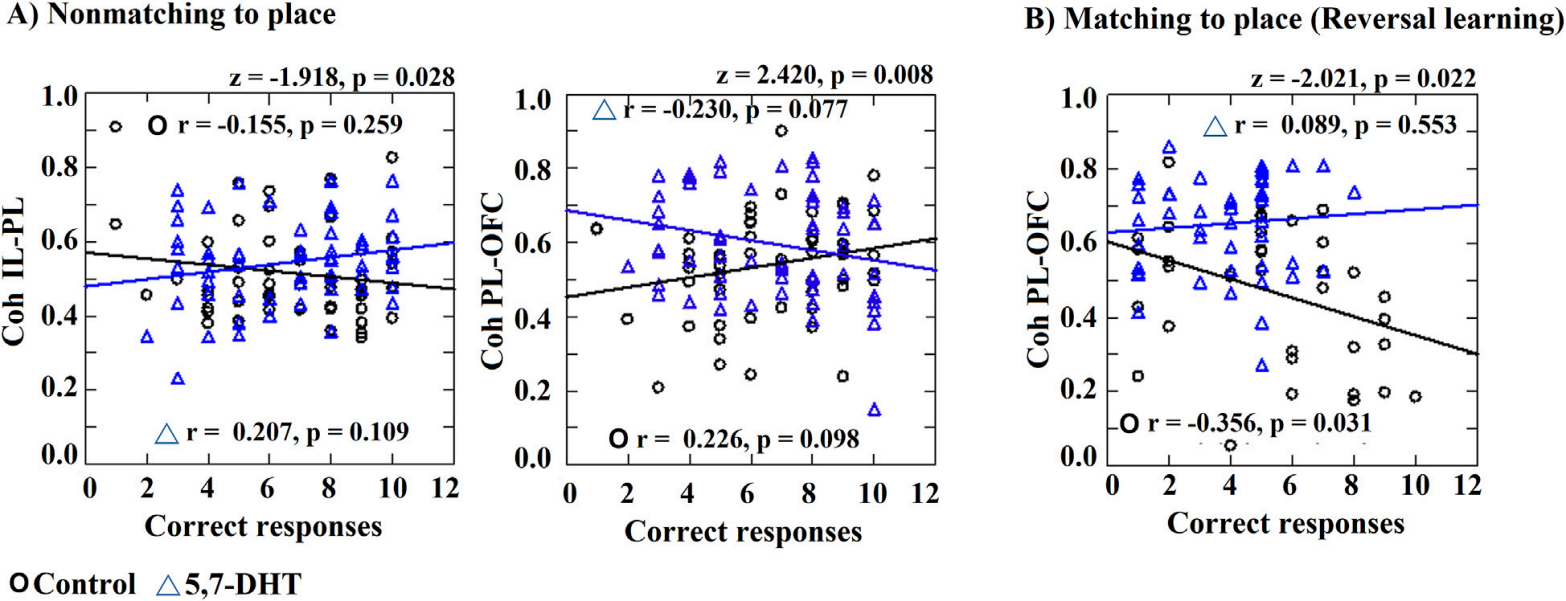
Correlation index between coherence and number of correct responses. Significant correlations or different correlations compared between the two groups are shown. **(A)** Non-matching-to-place IL-PL coherence and PL-OFC coherence. **(B)** Matching-to-place, PL-OFC coherence correlations with the number of correct responses. Pearson correlation and differences between correlation index (z values). Circles: control group and triangles: 5,7-DHT group. *p* < 0.05.

The between-group comparison of correlation values obtained in the non-matching-to-place task (first rule) showed significant differences for IL-PL (z = −1.918, *p* = 0.028) and PL-OFC (z = 2.420, *p* = 0.008) (Figure 8A).

In the reversal task, the correlation between PL-OFC coherence and the number of correct responses was significant and negative for the control group (r = −0.356, *p* = 0.031). No other significant correlation was observed in the control group or the 5,7DHT group. The correlation index comparisons between groups showed a significant difference in the PL-OFC correlation values (z = −2.021, *p* = 0.022) (Figure 8B).

## 4 Discussion

Several studies have highlighted the role of serotonin in modulating different behavioral components in different prefrontal subregions. For instance, Miyazaki et al. (2020) reported that serotoninergic activation in the OFC successfully promoted waiting with a fixed delay in reinforcement for future rewards, whereas when the delay was uncertain, serotoninergic activation in the mPFC promoted waiting for reinforcement. In addition, a stronger role of serotonin modulation in the extinction of previously learned behaviors, involving the IL cortex, and any influence on the control of conditioned fear, linked to Pl cortex functioning, have been observed (Lopez-Terrones et al., 2022; Bouton et al., 2021). Thus, different studies have shown that the effect of serotonergic manipulation on the prefrontal cortex depends on the targeted cortical subregion and the cognitive demands during behavioral tasks. Evidence indicates that reduction in prefrontal serotonin levels does not impair the acquisition of a response strategy; however, it does impair the change in previously learned strategies (Clarke et al., 2004). Thus, a deficiency occurs when a change in the task context requires the inhibition of a previously learned response and the acquisition of a new response (Alsio et al., 2021; Clarke et al., 2007; Roberts, 2011).

Our results are in accordance with the involvement of serotonin in the inhibition of a previously learned response, as well as with studies by Clarke et al. (2004), Clarke et al. (2007), and Alsio, Lehmann (Alsio et al., 2021), in which the reduction of serotonin levels in the OFC resulted in a delay in reversal learning without any effect on forward learning. However, when the reduction in serotonin level was general to the entire brain, the number of training sessions required to learn both visual discrimination and reversal learning increased (Alsio et al., 2021). Thus, the modulatory action of serotonin on prefrontal processes is prominent when behavioral tasks require strong participation of the PFC, whereas the learning process is affected only by serotonin depletion when it extends to brain regions other than the PFC.

The planning of response strategies to adapt behavior to changes in environmental contingencies is highly dependent on prefrontal function and is hindered by specific serotonergic reduction. In line with this, the current findings suggest that reducing prefrontal serotonin levels leads to a deficit in spatial reversal learning without affecting the learning process *per se* (evident in the learning of the first rule and in the decrease in the number of correct responses in the reversal task achieved by the serotonin-depleted animals); however, it impairs the inhibition of the first response and consequently the expression of the second response, causing a severe delay in the efficient performance of the second rule. Consistent with this, OFC and mPFC serotonin depletion caused by inescapable stress in rat models produces deficient reversal learning, which is restored by citalopram and vortioxetine treatments (Wallace et al., 2014; Natarajan et al., 2017; Danet et al., 2010; Lapiz-Bluhm et al., 2009). The endophenotype of low serotonergic activity due to low OFC tone predisposes rats to inflexibility and perseveration (Barlow et al., 2015).

Prefrontal serotonin exerts an inhibitory effect on pyramidal neuronal activity and a desynchronizing effect on hippocampal theta activity (Puig et al., 2005; Vertes and Kocsis, 1997); hence, a decrease in prefrontal serotonin would lead to an increase in theta power. According to the test trials, serotonin depletion resulted in a strong increase in theta power in the PL and OFC cortices, both in learning and reversal, and a weak increase in the IL theta power. Lopez-Terrones et al. (2022) indicated a strong inhibitory effect on IL compared to PL for serotonin released by dorsal raphe stimulation. The present results indicate a weak increase in the IL theta power and a strong increase in the PL and OFC theta power after serotonin depletion, possibly indicating stronger disinhibition in the PL and OFC. The three major differences with the present experiment are the behavioral condition (the rats were performing a reversal task), serotonergic manipulation (serotonin was reduced simultaneously in mPFC and OFC), and the type of recording (theta activity, which mainly reflects synaptic activity *versus* pyramidal neuron firing). These differences could account for the minimal effect observed on IL power and mPFC coherence (IL-PL) and the strong increase in theta power in the OFC and PL, along with the increased OFC-mPFC coherence observed in the present work. In addition, the stronger activation of the OFC linked to a delay in reversal learning observed here is in line with evidence that following a specific infusion of 5,7-DHT, mPFC serotonin depletion did not impair reversal learning, whereas OFC serotonin depletion impaired reversal learning (Alsio et al., 2021), while part of the increase in theta power in the PL would be related to the role of this region in spatial working memory (O'Neill et al., 2013), a component of the task unaffected by serotonin depletion. IL activity is related to emotional processing and stress-generated depression-like responses, whereas no effects on cognition have been observed after serotonergic manipulation in this region (Lopez-Terrones et al., 2022; Lopez-Terrones et al., 2023; Gasull-Camos et al., 2018). Thus, the minor effect on the IL would be related to the weak involvement of this region during cognitive processing compared with the PL, which is related to spatial working memory and the OFC implied in reversal learning.

Lopez-Vazquez et al. (2014) observed that septal serotonergic depletion facilitated spatial working memory assessed in an eight-radial-arm maze and increased the expression of concurrent hippocampal theta activity. In addition, Altman et al. (1990) and Gutierrez-Guzman et al. (2011) have reported that hippocampal serotonin depletion facilitates place learning. Thus, power increases related to the learning and reversal tasks were assessed by intragroup comparisons. The two groups showed an increase in IL power with the number of correct responses in the learning of the first rule, whereas in PL, only control animals had more correct responses that correlated with higher power. No significant correlation was observed in the serotonin-depleted group. This result indicates a strong effect of serotonin modification on PL than IL; however, as learning of the first rule was not different between groups, it is difficult to attribute this correlation to learning.

In reversal learning, the serotonin-depleted group showed a positive correlation between theta power in the OFC and the number of correct responses, in contrast to the control group, which showed no significant negative correlation. Under control conditions, an increase in theta power related to efficient performance did not occur. In fact, increased theta power in the serotonin-depleted group was related to deficient reversal learning. In addition, PL-COF coherence was negatively correlated with the number of correct responses in the reversal task (matching-to-place), possibly indicating that the disengagement of this structure is required for the inhibition of the first response and expression of the second response.

The higher theta power may be a result of the failure in inhibitory control after serotonin depletion, which was dependent on the sub-region of the PFC and task condition. This differential effect depends on the characteristics of serotonergic innervation and the serotonergic receptors expressed in each region, which may result in functional dissociation (Boulougouris et al., 2007; Goncalves et al., 2009; Commons, 2016; Leiser et al., 2015; Linley et al., 2013). Serotonin inhibits mPFC pyramidal neurons directly through 5HT1_A_ receptors and indirectly through the excitation of interneurons mediated by 5HT_3_ receptors. In addition, pyramidal neurons are excited by serotonin via 5HT2_A_ and 5HT2_C_ receptors, whereas GABAergic neurons express inhibitory 5HT1_A_ and excitatory 5HT2_A_ receptors (Puig and Gulledge, 2011; Pompeiano et al., 1992; Morales and Bloom, 1997; Pompeiano et al., 1994). In addition, the OFC expresses a high density of excitatory 5HT_7_ receptors which are related to reversal learning, specifically in the modulation of perseverative responses (Hrnjadovic et al., 2021; Nikiforuk et al., 2015). Although the principal effect of serotonin is complex and depends on the receptors, cortical layer, and type of neuron, its inhibitory effect is prominent (Puig et al., 2005) and is compatible with the increased theta activity observed after serotonin depletion.

Coherence is an index of functional coupling between the cerebral structures involved in cognitive processing (Fell and Axmacher, 2011). Under control conditions, high coherence between the mPFC and OFC occurred during the trials in which the information about the change in the environment was updated; subsequently, the coherence decreased below the initial training values. Increased coherence between the hippocampus and the PFC has been related to successful performance in a spatial alternation task during spatial decision-making (Tavares and Tort, 2022) during successful spatial working memory (O'Neill et al., 2013; de Mooij-van Malsen et al., 2023).

Similar to that observed in theta power, the coherence between the OFC-PL and OFC-IL was higher in serotonin-depleted animals both in learning and reversal (see Figure 6). Moreover, it is evident that groups have distinct coherence patterns during training. The non-significant interaction between block and condition in these cortices was principally due to the similar values in baseline and test trials. Paired comparisons of test trials were significant for the reversal learning, when the OFC-PL and OFC-IL coherences showed a sharp increase from B1 to B6 and reduction from B6 to B8 and B10. These changes match with the time of the training when the animals change their response from the first rule (non-matching-to-position) to the second rule (matching-to-position) to attain the 50% of efficiency. In B8 and B10, the control animals attained 70% efficiency, and the coherence decreased below the values at the start of the training. Similarly, increased cortical–hippocampal theta synchronization has been linked to increased performance efficiency during spatial working memory tasks (O'Neill et al., 2013; Benchenane et al., 2010). This evidence indicates that increasing prefrontal medial–orbitofrontal functional coupling is required for successful performance of prefrontal-dependent reversal tests. Once the animals acquire the new rule, the mPFC-OFC coherence was observed, possibly indicating that communication is not needed to display the adapted behavior. In contrast, the coherence of the serotonin-depleted group was high in the initial block of reversal, and the values were similar on blocks 1, 6, and 8, which finally started to decrease until block 10, when the rats reached 50% efficiency, similar to the control rats in block 6. Thus, increased coherence is necessary to inhibit the first response learned and for the acquisition of the new rule, both in control and 5-HT-depleted rats; the temporal course was slow in serotonin-depleted rats, which started with high coherence values. The increase in coherence in control rats could be related to both the inhibition of the previous response and the acquisition of the second rule, whereas in serotonin-depleted conditions, a state of increased coherence works as the interference, impairing reversal learning, as a type of noise signal that hinders the reversal learning-related increase in coherence.

Although no other studies have assessed the impact of serotonin depletion on prefrontal theta activity related to learning, there has been an increase in coherence reported in other cognitive circuits. After hippocampal or septal serotonin depletion, which leads to an increase in septo-hippocampal and septo-mammillary theta-band EEG coherence during spatial learning in the Morris water maze, place learning facilitation occurred (Gutierrez-Guzman et al., 2011; Gutierrez-Guzman et al., 2017). However, after supramammillary serotonin reduction, coherence was modified in the septo-hippocampal circuit, with higher intrahippocampal coherence and lower septo-hippocampal coherence, resulting in a strong deficit in spatial learning (Hernandez-Perez et al., 2015). Thus, serotonin appears to tune the fine coupling between the circuits that sustain memory and cognitive processing. A failure of the inhibitory action of serotonin on the PFC in serotonin-depleted rats may cause poor inhibition of the previously learned response or the inability to learn the new rule. Although functional coupling among the prefrontal areas occurred in both groups after learning the first rule, only the control group showed a reduction in IL-OFC and PL-OFC coherence in the final phases of reversal learning. This reduction possibly allows the expression of differential processing underlying reversal learning. Accordingly, Cano-Colino et al. (2013) proposed in a computational model that modifications in the prefrontal serotonin underlying working memory tests can change the attentional control of stimuli-guiding behavioral responses. In this view, a reduction in prefrontal serotonin facilitates attention to stimuli that guide behavior and reduces attention to distractors, thereby favoring working memory. Thus, during reversal learning, serotonin depletion favors a perseverant response toward the stimuli that guide initial learning, impairing the ability to shift from cognitive–behavioral responses according to the first rule toward different behavioral–cognitive responses, now dependent on new guiding stimuli, according to the second rule.

Although some studies have focused on the interaction between the prefrontal and hippocampal regions during reversal learning, studies monitoring the coupling between the mPFC and OFC during these types of tasks are lacking. The firing of prefrontal OFC neurons and hippocampal theta activity during acquisition were analyzed by Riceberg et al. (2022) who showed that a decay after the learning criterion was attained. Moreover, Mau et al. identified the formation of neuronal ensembles with stable activation strength when an animal learned a memory task. From this perspective, when reversal learning occurs, a change in active ensembles can occur because of the uncoupling of previously active ensembles and the formation of new ones (Mau et al., 2020). In conjunction with previous studies, the present results suggest that reversal learning, as an expression of behavioral flexibility, requires specific modulation of theta activity by the serotonergic system as serotonin plays a role in mediating behavioral inhibition (Cools et al., 2008; Logue and Gould, 2014). Such modulation involves fine-tuning the power and coherence of prefrontal subregions during different phases of the reversal test, allowing for expression of adaptive responses to changes in environmental contingencies.

Serotonin and dopamine have a close relationship as modulators of the prefrontal cortex, acting as opposite systems in cognitive modulation; thus, serotonin reduction facilitates dopamine-dependent learning (Olvera-Cortes et al., 2008). The present results indicate an impairment in reversal learning after serotonin depletion by the action of the neurotoxin 5,7-DHT; thus, it is possible to assume that dopamine modifications would take place after serotonin depletion. However, Sakaue et al. (2000) injected 5,7-DHT into the lateral ventricles and measured the serotonin content in PFC 14 days later. The findings indicated that prefrontal serotonin level decreased by 96%–97% without any impact on dopamine or noradrenaline (Sakaue et al., 2000). In an earlier work, Reader and Gauthier (1984), also injected 5,7-DHT intraventricular and measured the levels of 5-HT, norepinephrine, and dopamine in different cerebral areas 15 days after the administration of the neurotoxin. The results of radioenzymatic assays showed a reduction in serotonin levels in the PFC without any effect on the other two catecholamines (Reader and Gauthier, 1984). In order to protect noradrenergic terminals, in the first work, desipramine was administered 30 min before the neurotoxin infusion, and in the second work, imipramine was administered 1 h before infusion of the 5,7-DHT. In a more recent work, Alsio et al. (2021) infused 5,7-DHT to the mPFC or OFC 30 min after desipramine administration (ip.) and observed reductions of 90% in mPFC 5-HT and 80% in OFC 5-HT, without effects on dopamine and norepinephrine levels. In the present work, desipramine was administrated 30 min before infusion of 5,7-DHT, and the neurotoxin infusion did not result in significant differences in dopamine and DOPAC levels (Figure 2A, right). Thus, the changes in reversal learning observed here could be attributed to the reduction in serotonin levels. The role of serotonin in regulating impulsivity (Desrochers et al., 2022) suggests that the deficient reversal learning observed here may have been associated with higher impulsivity. However, no differences in the latencies for choosing the arm in the test trials were observed between groups. In fact, a trend for longer latencies was observed in the serotonin-depleted group. This trend was related to a longer time spent in the zone where the decision of which arm to enter is taken. Thus, the failure of reversal learning was not related to impulsivity.

Obsessive compulsive disorders (OCD), post-traumatic stress disorders (PTSD), schizophrenia, gambling disorders, and stimulant use disorder are pathologies that are related to both with impaired behavioral flexibility and serotonergic alterations (Vaghi et al., 2017; Waltz and Gold, 2007; Verdejo-Garcia et al., 2015; Marx et al., 2021; Kish et al., 2009; Kishi et al., 2009; Hasselbalch et al., 2007; Lopez-Figueroa et al., 2004). Our results are in accord with a deficient reversal learning possibly related with the impaired change in the expression of a new response after the learning of a first response in conditions of reduced levels of prefrontal serotonin. Possibly as an expression of impaired behavioral inhibition, a key component of cognitive control (Friedman and Robbins, 2022). Changes in prefrontal neural activity, as observed in the present results, may occur before appearance of behavioral signs in the different pathologies involving alterations in the serotonergic system and impaired behavioral flexibility.

## Data Availability

The raw data supporting the conclusions of this article will be made available by the authors, without undue reservation.
